# Epidermal Growth Factor Like-domain 7 and miR-126 are abnormally expressed in diffuse Systemic Sclerosis fibroblasts

**DOI:** 10.1038/s41598-019-39485-8

**Published:** 2019-03-14

**Authors:** Vasiliki Liakouli, Paola Cipriani, Paola Di Benedetto, Noemi Panzera, Piero Ruscitti, Ilenia Pantano, Onorina Berardicurti, Francesco Carubbi, Filomena Esteves, Georgia Mavria, Francesco Del Galdo, Roberto Giacomelli

**Affiliations:** 1 Department of Biotechnological and Applied Clinical Science, Rheumatology Unit, School of Medicine, University of L’Aquila, Delta 6 Building, Via dell’Ospedale, 67100 L’Aquila, Italy; 2 Leeds Institute of Cancer and Pathology, University of Leeds, Leeds, UK; 3 Signal Transduction and Tumor Microenvironment Group, Leeds Institute of Cancer and Pathology, University of Leeds, Leeds, UK; 4 Division of Rheumatic and Musculoskeletal Diseases, Leeds Institute of Molecular Medicine, University of Leeds, Leeds, UK

## Abstract

Systemic sclerosis (SSc) is characterized by microangiopathy with impaired reparative angiogenesis and fibrosis. Epidermal Growth Factor Like-domain 7 (EGFL7), firstly described in endothelial cells plays a pivotal role in angiogenesis. Fibroblasts (FBs) are involved in vascular remodeling, under physiological and pathological conditions. In this study, we investigated: (i) the expression of EGFL7 and its miR-126 in patients affected by diffuse cutaneous SSc (dcSSc); (ii) the ability of Transforming Growth Factor-beta (TGF-β) to modulate EGFL7 expression; (iii) the ability of EGFL7 to modulate COL1A1 expression and proliferation/migration, and (iv) the functional role of EGFL7 on angiogenesis. Patients were divided in 2 subsets: patients fulfilling the classification criteria in less than one year from Raynaud’s Phenomenon onset (Early Onset Subset–EOS), and all the others (Long Standing Subset–LSS). We show that EGFL7 expression is increased in EOS dcSSc skin and cultured FBs. EGFL7 is inducible by TGF-β on Healthy Controls (HC) FBs but not in SSc-FBs. EGFL7 decreases COL1A1 expression in EOS SSc-FBs while EGFL7 silencing up-regulates COL1A1 expression. EGFL7 promotes migration/invasion of EOS SSc-FBs but not proliferation. Finally, SSc-FBs, partially inhibit angiogenesis in organotypic coculture assays, and this is reversed by treatment with human recombinant (rh)EGFL7. We conclude that EGFL7 and its specific microRNA miR-126 may be involved in the pathogenesis of SSc vasculopathy and fibrosis.

## Introduction

Systemic sclerosis (SSc) is an autoimmune disease characterized by a widespread microangiopathy, autoimmunity and abnormal fibrosis of the skin and internal organs^1^. Microangiopathy is characterized by a reduced capillary density associated with irregular and chaotic architecture, which leads to chronic tissue hypoxia. Angiogenesis, the formation of new capillaries from pre-existing vessel, is primarily driven by tissue hypoxia and occurs under physiological and pathological conditions. Despite the presence of hypoxia, there is no evidence of a compensatory mechanism that promotes angiogenesis in SSc^2–4^.

Angiogenesis, is a highly complex process, requiring dynamic, temporally and spatially interactions among ECs, soluble pro- and anti-angiogenic growth factors, extracellular matrix (ECM), adhesion molecules and proteolytic enzymes. The process depends on the local ECM, which plays two major roles: provides a structural framework and acts as source of several pro and anti-angiogenic factors through regulation of their localisation and bio-availability^5^.

EGFL7, expression which was first described in endothelial cells (ECs) plays a pivotal role in the development of the vascular system^6,7^. During embryogenesis, EGFL7 is highly expressed in ECs, while postnatally its expression markedly decreases. On the other hand, expression is up-regulated during reparative angiogenesis^8–10^ when EGFL7 modulates ECM rigidity and promotes cell migration and invasion through inhibition of mature elastic fibers^11^, thus preventing premature vessel stabilization and allowing capillary sprouting^6^. Although initially it was thought that EGFL7 is specifically expressed by ECs, recently it has been shown that EGFL7 is differentially expressed in normal adult tissues, and overexpressed in epithelial tumor tissues^12^. Lately members expression of EGF-like family such as EGFL6 and EGFL7 has been reported in osteoblastic/osteoclastic cells, and where it may play a vital role in the cross-talk with ECs, thus modulating angiogenesis during bone remodeling^13,14^. FBs are involved in physiological and pathological angiogenesis through secretion of different ECM-related molecules, such as collagens, fibronectin, heparan sulfate and proteoglycans^15–17^, thus playing an active role in the organization of the provisional matrix that supports angiogenic growth. During pathological angiogenesis, FBs become activated and secrete angiogenic growth factors such as Vascular Endothelial Growth Factor (VEGF), Platelet Derived Growth Factor (PDGF) and Stromal cell-Derived Factor-1 (SDF-1)^18,19^.

The role of EGFL7 in the adult mammalian vasculature is not precisely understood and is complicated by the presence of a microRNA, miR-126, within the EGFL7 gene^20–22^. MicroRNAs, a class of about 22 nucleotide long, non-coding RNAs, are regulators of gene expression by functioning as endogenous inhibitors of the process of translation^23,24^. Studies have shown that microRNAs are involved in the pathogenesis of autoimmune disorders^25,26^ and malignancies^27^. miR-126 is highly expressed in ECs, and mice lacking miR-126 but expressing EGFL7 display embryonic lethality caused by loss of vascular integrity^20–22^. However, the presence of miR-126 within the sequence of the EGFL7 gene is predicted to negatively regulate EGFL7 expression in FBs, and EGFL7 has been identified as a potential miR-126 target by bioinformatics approaches. In a recent study it was shown that the binding site of miR-126 lies within the 3′-UTR of EGFL7*;* and that the EGFL7 gene is down-regulated in human lung cancer cell lines^28^.

To assess the hypothesis that EGFL7 is expressed on dcSSc-FBs and involved in SSc pathogenesis, we investigated: (i) the expression of EGFL7 and its miR-126 in the skin and cultured FBs isolated from SSc patients fulfilling the 2013 classification criteria for SSc^29^, (ii) the ability of TGF-β, the major pro-fibrotic cytokine in SSc, to modulate EGFL7 expression in FBS: (iii) the role of EGFL7 in modulating COL1A1 expression, thus influencing ECM rigidity and new vessel development and (iv) the functional role of EGFL7 in the process of angiogenesis in organotypic endothelial-fibroblast co-cultures.

## Results

### EGFL7 expression is increased in EOS dcSSc skin biopsies compared to HC and downregulated in LSS dcSSc

EGFL7 expression in skin biopsies from SSc patients and HC was evaluated by IHC. The CD34 marker was used in double IHC to immunostain ECs lining blood vessels, and the α-SMA marker was used to immnunostain pericytes and myofibroblasts.

**Figure 1:**
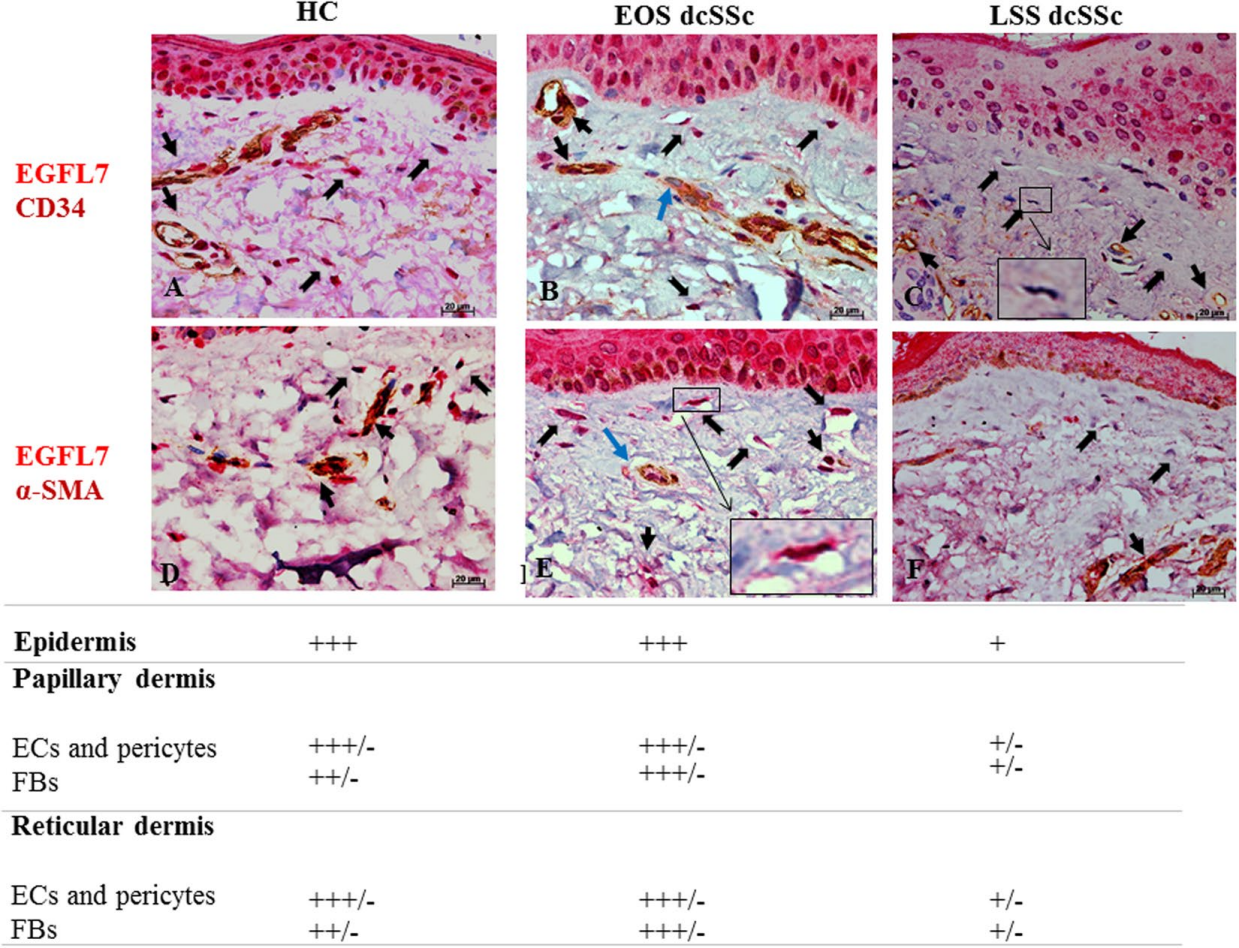
EGFL7 immunostaining in HC and dcSSc skin. (**A**,**D**) In HC skin, strong EGFL7 expression was detected in ECs and pericytes of vessels (black arrows), in fibroblasts of papillary and reticular dermis (black open arrows), and cells of the basal layer of the epidermis and keratinocytes. (**B**,**E**) In EOS dcSSc skin, strong EGFL7 expression was detected in ECs and pericytes of vessels (black arrows), in fibroblasts of papillary and reticular dermis and epidermal (black open arrows), cells of the basal layer of the epidermis and keratinocytes as in the HC skin. ECs of the most vessels were strong immunostained for EGFL7. However, ECs of a few vessels displayed a weaker immunostaining intensity for EGFL7 (blu arrows). Inset shows EGFL7 positive fibroblast (**C** and **F**) In LSS dcSSc skin, the remaining microvessels displayed a weak (or absent) immunostaining for EGFL7. Furthermore, fibroblasts of papillary and reticular dermis and cells of the basal layer of the epidermis and keratinocytes displayed a weak (or absent) immunostaining for EGFL7. Inset shows EGFL7 weak positive fibroblast. Semiquantitative scoring was performed independently by 2 blinded observers, based on the observation of immunostained skin sections. Scores are defined as follows: +++ intense staining; +++/− intense staining but not homogeneous positive staining; ++ moderate staining; + weak staining; +/− weak staining but not homogeneous positive staining.

In HC skin, strong EGFL7 expression was detected in ECs and pericytes associated with blood vessels, in fibroblasts of papillary and reticular dermis, and cells of the basal layer of the epidermis and keratinocytes (Fig. 1A,D).

A similar pattern of strong EGFL7 expression was detected in EOS dcSSc skin, however a number of vessels were identified that displayed weaker EGFL7 immunostaining in ECs (Fig. 1B).

In LSS dcSSc skin, the small number of microvessels identified displayed weak, or no immunostaining for EGFL7. Furthermore, fibroblasts of papillary and reticular dermis and cells of the basal layer of the epidermis and keratinocytes also displayed weak, or no EGFL7 immunostaining in LSS dcSSc skin (Fig. 1C,F).

### EGFL7 mRNA and protein expression levels are increased in EOS dcSSc FBs compared to HC

**Figure 2:**
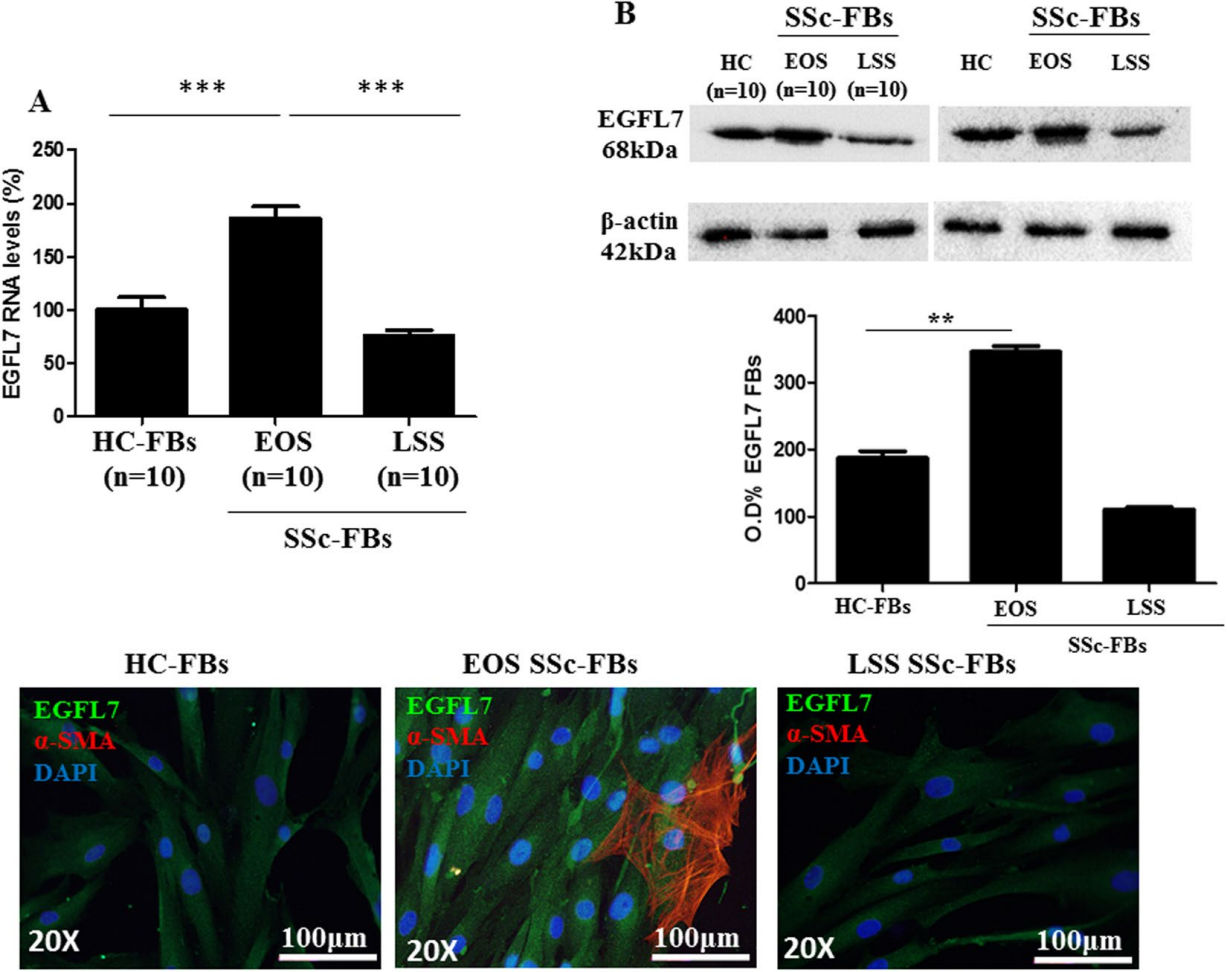
Abnormal expression of EGFL7 mRNA and protein levels in FBs of SSc patients (**A–C**). FBs isolated from the skin of EOS SSc patients, express a statistically significant higher levels of EGFL7 mRNA, when compared to HC and LSS SSc-FBs (^***^p = 0.0001). The experiments were performed in triplicate for each patient and HC. Bars represent mean values ± SEM (N = 10 for each group) (**A**). 18 s gene served as the control. WB analysis of EGFL7 (**B**). These results confirm at protein level the results of qRT-PCR analysis. Blots were stripped using Re-Blot Plus Western Blot recycling kit (Chemicon International, USA) and re-probed with anti-mouse IgG β-actin antibody (Sigma-Aldrich, USA) to confirm similar loading of the gels and efficiency in electrophoretic transfer. The experiments were performed in triplicate for each patient and HC. Full length blot is represented in Supplementary Fig. 4. Immunoreactive bands were acquired by chemidoc (ImageLab). Densitometric analysis of the bands was performed using ImageJ software (NIH; online at http://rsbweb.nih.gov/ij) (**C**). IF on cultured FBs at 60% of confluence. Negative controls were obtained by omitting the primary antibody.

We examined the levels of EGFL7 mRNA and protein levels in isolated SSc-FBs obtained from both EOS and LSS patients and compared them to those observed in HC. A statistically significant difference in EGFL7 mRNA levels was found between the SSc-FBs and HC-FBs (Fig. 2A). In particular, FBs isolated from the skin of EOS SSc patients, expressed approximately 2-fold higher levels of EGFL7 mRNA, when compared with HC, and LSS SSc-FBs (p < 0.0001).

Evaluation of EGFL7 protein expression levels using WB analysis showed that the protein expression profiles paralleled the mRNA levels (Fig. 2B). FBs isolated from EOS SSc skin, expressed approximately 1.8-fold higher EGFL7 protein levels compared to HC- and LSS SSc-FBs.

The expression and subcellular localization of EGFL7 in dermal FBs was also investigated by IF. We found that EGFL7 was mainly localized in the perinuclear cytoplasm of HC- and SSc-FBs, independently of the onset of the disease (Fig. 2C).

### EGFL7 is inducible by TGF-β on HC-FBs

**Figure 3:**
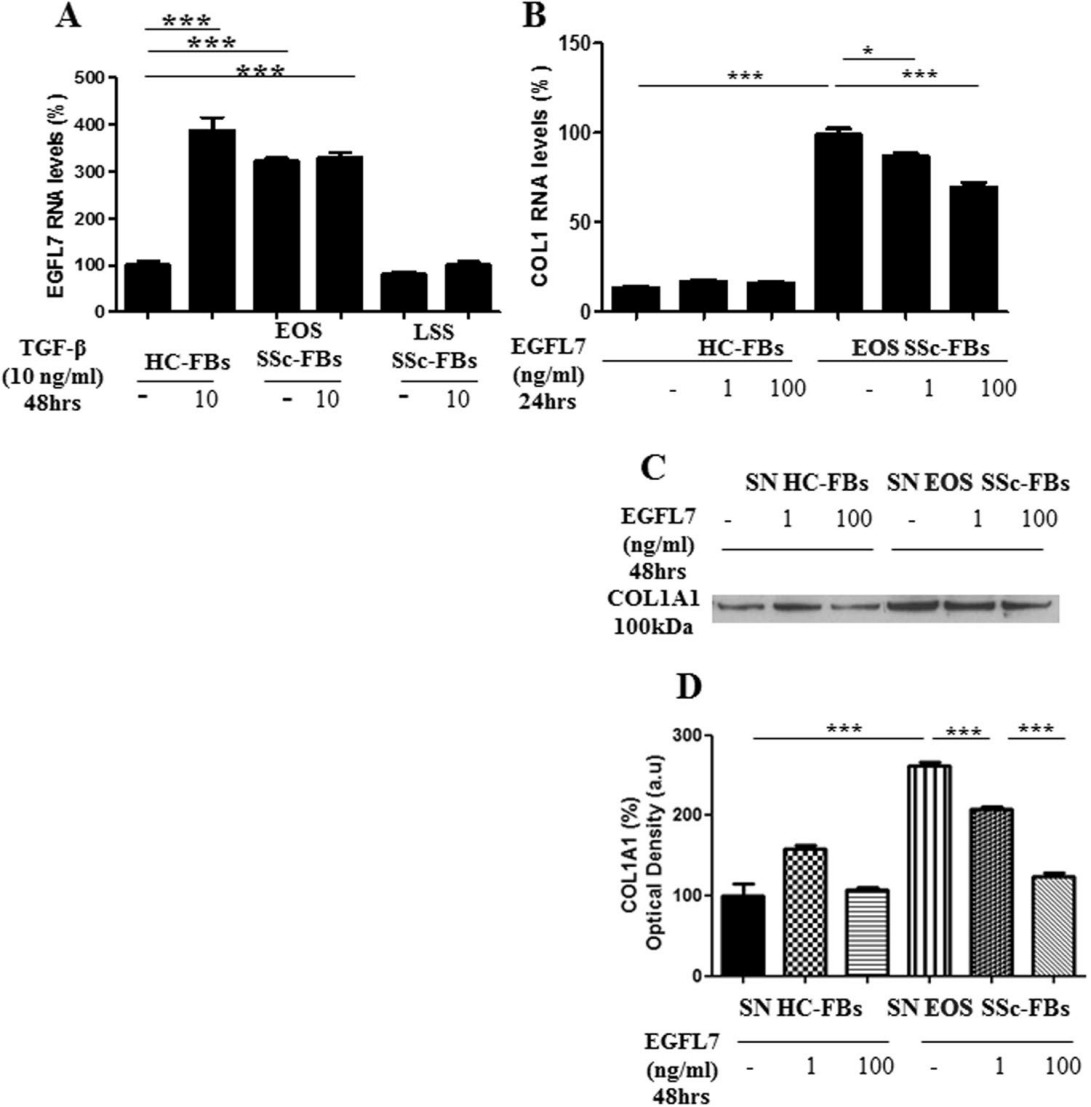
EGFL7 is inducible by TGF-β on HC-FBs and decreases the expression of COL1A1 (**A–G**). The experiments were performed in triplicate. Bars represent mean values ± SEM before and after stimulation with 10 ng/ml of TGF-β for 48 hrs. The absence of rh proteins from the medium of stimulation was considered as a negative control. HC-FBs, after stimulation by TGF-β, expressed significantly higher levels of EGFL7 RNA, when compared to untreated HC-FBs (^***^p < 0.0001). On the contrary, TGF-β treated EOS SSc-FBs did not show any difference in the EGFL7-RNA levels, when compared to the untreated EOS SSc-FBs. No effect was observed, on the EGFL7 RNA levels for the TGF-β treated and untreated LSS SSc-FBs (**A**). The levels of TGF-β in untreated HC-FBs were set to 100% and all the results were normalized to this value. After EGFL7 stimulation, we observed a dose-related fashion decrease of the expression of COL1A1 (^*^p = 0.018; ^***^p = 0.0001 for RNA levels), both in RNA and protein levels, in SSc-FBs. On the contrary, when HC-FBs were treated with the same rhEGFL7 concentrations, the expression of COL1A1 protein did not change at any concentration of EGFL7 stimulation (**B,C**). Full length blot is represented in Supplementary Fig. 2. Densitometric analysis of the bands was performed using ImageJ software (NIH; online at http://rsbweb.nih.gov/ij) (**D**).

Cells were treated with rhTGF-β and total mRNA was extracted and analyzed by qRT-PCR. Following stimulation by TGF-β, HC-FBs expressed 4-fold higher levels of EGFL7 mRNA when compared with untreated HC-FBs (p < 0.0001). On the contrary, TGF-β treated EOS SSc-FBs did not show any difference in the EGFL7 mRNA levels, when compared with the untreated EOS SSc-FBs (p = 0.06). Interestingly, after treatment TGF-β treated HC-FBs reached equivalent EGFL7 mRNA levels to TGF-β treated EOS SSc-FBs (p = 0.06). No effect was observed on the EGFL7 mRNA levels both in TGF-β treated and untreated LSS SSc-FBs (p = 0.06). EGFL7 mRNA levels in LSS SSc-FBs TGF-β treated or untreated, were similar to those detected in untreated HC-FBs (p = 0.94) (Fig. 3A).

### EGFL7 suppresses COL1A1 expression in EOS dcSSc-FBs

HC- and SSc-FBs were treated with increasing concentrations of rhEGFL7 and total RNA was extracted and analyzed by qRT-PCR. Figure 3B shows that treatment with rhEGFL7 downregulated COL1A1 mRNA levels in SSc-FBs in a dose-dependent manner (p = 0.01 and p = 0.0001), although COL1A1 mRNA levels did not decrease to those of HC-FBs. Consistent with the notion of EGFL7 suppressing COL1A1 expression, HC-FBs mRNA levels were unresponsive to any applied concentration of EGFL7 (p = 0.13) (Fig. 3BD). Secreted COL1A1 protein levels detected by WB in supernatants of cultured HC- and SSc-FBs paralleled the mRNA levels (Fig. 3C) (p < 0.0001).

**Figure 4:**
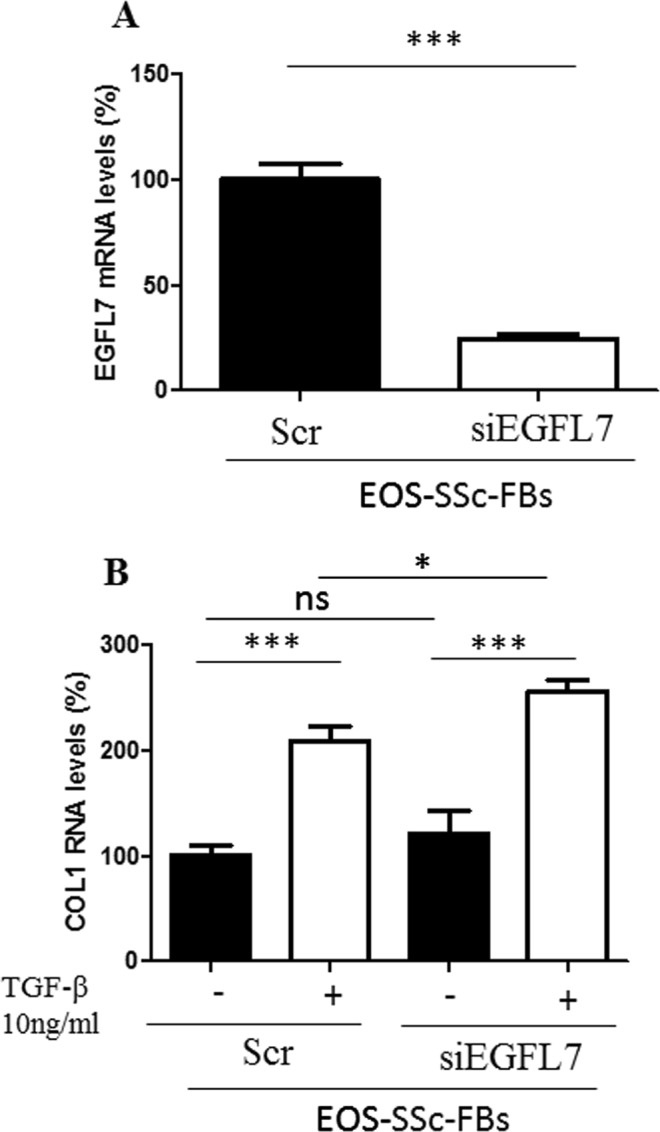
EGFL7 knockdown in HC- and SSc-FBs up-regulates Col1A1 expression after TGF-β stimulation. SSc-FBs were transfected with specific EGFL7-siRNA (siRNA) or non-targeting siRNA (scr), and EGFL7 mRNA expression was evaluated by qRT-PCR analysis. The cells transfected with EGFL7-siRNA showed a decreased expression of EGFL7 mRNA levels when compared with cells transfected with scr siRNA (**A**). After TGF-β stimulation, the silenced cells show an overexpression of COL1A1, suggesting the anti-fibrotic role of EGFL7, confirming the effects observed in Fig. 3B. Each experimental condition was performed in triplicate. Bars represent mean values ± SEM.

We then investigated the effects of EGFL7 knockdown in SSc-FBs in the regulation of collagen. Figure 4 shows that there was efficient knockdown of EGFL7 in SSc-FBs transfected with EGFL7 siRNA, when compared with cells transfected with non-targeting siRNA (scr) (p < 0.0001). EGFL7 knockdown did not affect TGF-β driven stimulation of COL1A1 in SSc-FBs consistent with EGFL7 being involved in the suppression of COL1A1. Interestingly, there was further stimulation of COL1A1 with EGFL7 knockdown as in TGF-β stimulated SSc-FBs there was a significant 20% increase in COL1A1 mRNA levels by qRT-PCR analysis.

### EGFL7 promotes migration and invasion of SSc-FBs but not proliferation

**Figure 5:**
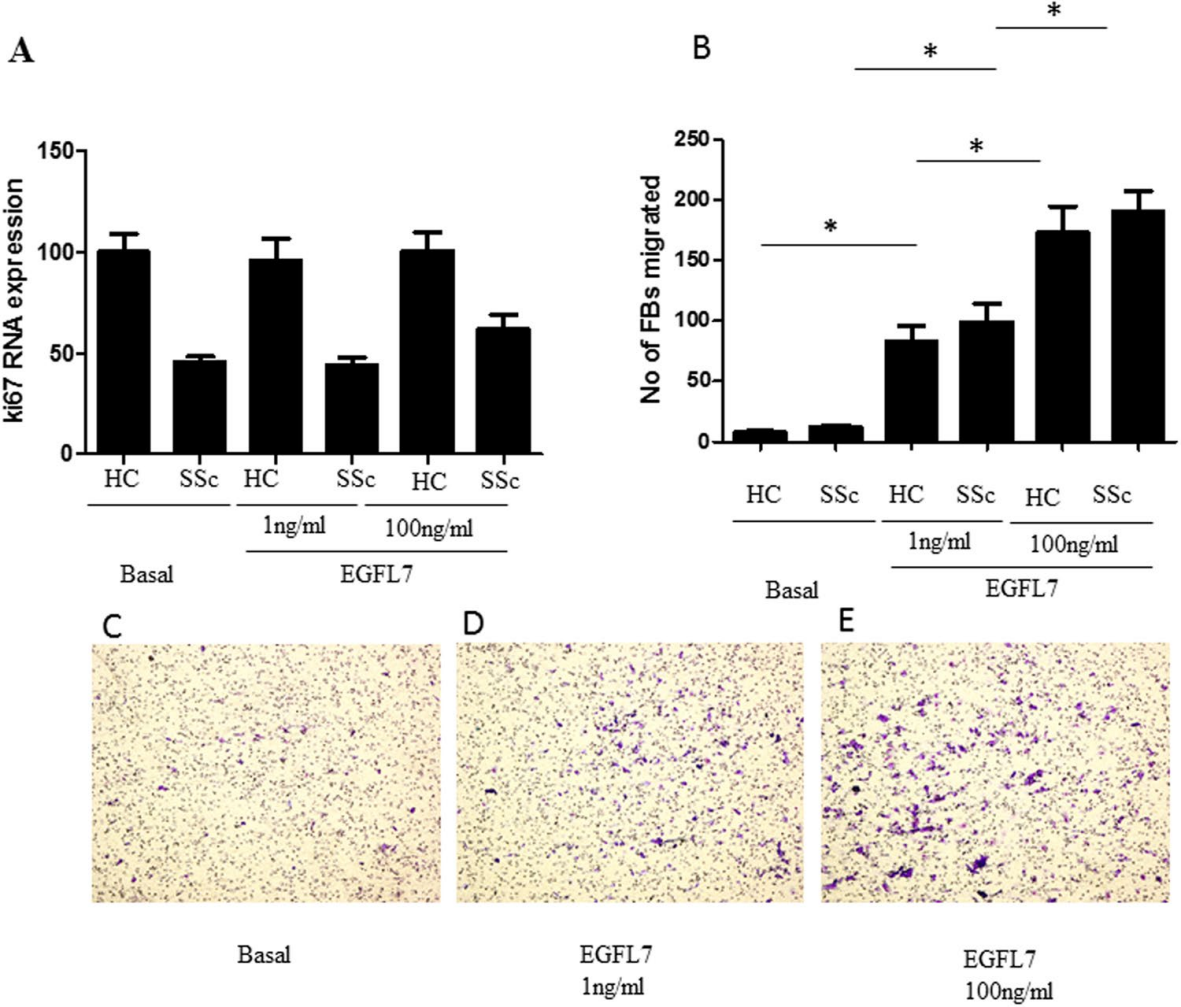
EGFL7 promotes migration and invasion but not proliferation of EOS dcSSc-FBs. At basal state EOS SSc-FBs, expressed a significantly lower ki67 transcript, when compared to that of HC. EGFL7 did not modulates the proliferation of treated HC- and EOS-FBs (**A**). Furthermore, we assessed the migratory ability and invasion of our cells and we found that HC- and EOS dcSSc-FBs migration and invasion were significantly induced by EGFL7 in a concentration dependent-manner (1–100 ng/ml) (p = 0.013 and p = 0.0044 and p = 0.0020) (**B**). All assays were independently performed in triplicate and repeated at least thrice. Bars represent mean values ± SEM. Representative photomicrographs show SSc-FBs migration at basal state (**C**), following stimulation with EGFL7 1 ng/ml (**D**) and EGFL7 100 ng/ml (**E**). Original magnification X20.

To assess the proliferative ability of HC- and SSc-FBs, we performed qRT-PCR ki67 as proliferative marker. Ki67 mRNA levels were unresponsive to any concentration of EGFL7 applied to the cultures (Fig. 5A). We then assessed the migratory ability and invasion of FBs and found that HC and EOS dcSSc-FBs migration and invasion were induced by EGFL7 in a concentration dependent-manner (1–100 ng/ml) without difference between SSc and HC (for EGFL7 1 ng/ml: p = 0.013 both for untreated HC- and SSc-FBs compared with treated HC- and SSc-FBs and for EGFL7 100 ng/ml: p = 0.0044 and p = 0.0020 for untreated HC- and SSc-FBs compared with treated HC- and SSc-FBs).

### miR-126 expression is decreased in cultured EOS SSc-FBs

**Figure 6:**
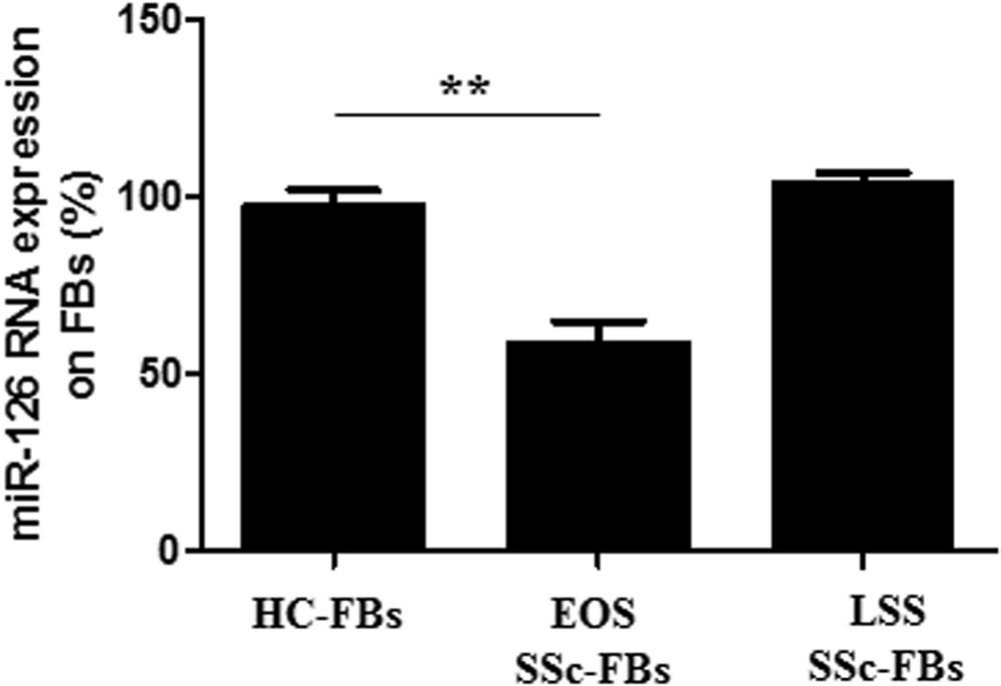
Abnormal expression of miR-126 in cultured SSc-FBs (**A**). FBs isolated from the skin of EOS SSc patients, express a statistically significant lower levels of miR-126, when compared to HC- and LSS FBs; p = 0.0001 (**A**). The expression levels of miR-126 HC-FBs were set to 100% and all the results were normalized to this value. RNU6B gene served as the control. For relative quantification, the comparative threshold cycle (Ct) method was used. The experiments were performed in triplicate, for each patients and HC. Bars represent mean values ± SEM.

We analyzed the expression of the miR-126 in both cultured HC- and SSc-FBs by qRT-PC. We showed that FBs isolated from the skin of EOS dcSSc patients, showed a 45% reduction of miR-126 mRNA levels, when compared with HC- and LSS dcSSc-FBs (p = 0.0001) (Fig. 6).

### EGFL7 restores defective angiogenesis by SSc-FBs in organotypic coculture assays

**Figure 7:**
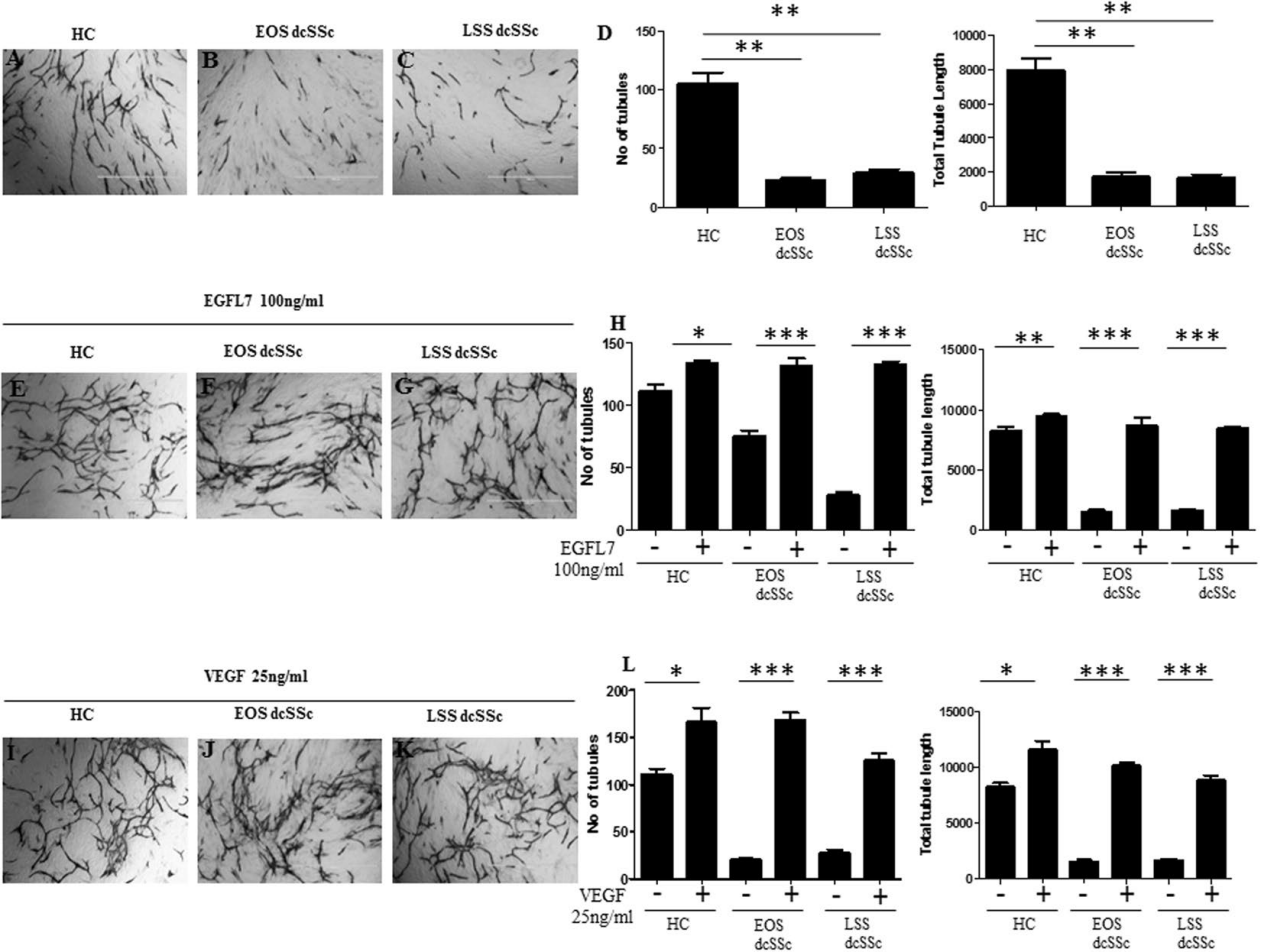
SSc-FBs are responsible for impaired angiogenesis in an organotypic co-culture assay system *in vitro* but EGFL7 restores angiogenesis. Angiogenesis was not affected when primary HC-FBs were co-cultured with HUVECS (**A**). Interestingly, when primary SSc-FBs were co-cultured with HUVECs, angiogenesis was impaired when compared with the tubule formation obtained from co-cultured HC-FBs with HUVECs (**B–C**). EGFL7 increases the tubule formation when added to the cultured medium (100 ng/ml) thus adding EGFL7 to the list of the pro-angiogenic molecules that rescue angiogenesis defects in SSc patients (**E–G**). VEGF (25 ng/ml) was used as positive control (**F–K**). Representative microscopic fields at 4X magnification from triplicate wells. Vascular junction and tubule number and total tubule length were analysed by Angiosys System (TCS CellWorks, USA) (**D**–**H**–**L**). Bars represent mean values ± SEM (measurment of 12 microscopic fields from triplicate wells). Statistical analysis was performed by unpaired and paired t test, two tailed. ^*^p < 0.05; ^**^p < 0.001; ^***^p < 0.0001.

Previous published work has shown that dermal SSc-FBs promote defective angiogenesis in an organotypic co-culture assay of angiogenesis^30^. When SSc-FBs are co-cultured with HUVECs, tubule formation was impaired when compared with tubule observed when using HC-FBs in coculture (Fig. 7A–D). The total number of tubules and their total length were statistically decreased by approximately 70% and 60%, respectively (Fig. 7D). Treatment with rhEGFL7 at 100 ng/ml of concentration, rescued the defect of angiogenesis driven by both EOS and LSS dcSSc-FBs in SSc-FBs/HUVECs co-cultures (Fig. 7E–G). In particular, the total number of tubes and their total length were statistically increased (3 and 4-fold, respectively) (Fig. 7). rhVEGF 25 ng/ml was used as a positive control (Fig. 7).

### EGFL7 serum levels are increased in the EOS and LSS dcSSc patients

The levels of secreted EGFL7 were determined in the sera of dcSSc patients and compared to those from HC. We found higher secreted EGFL7 levels in the sera of EOS and LSS dcSSc patients compared to HC (p = 0.006 and p = 0.0007 respectively) (Suppl. Fig. 1). Interestingly, secreted EGFL7 was somewhat higher in the sera of LSS dcSSc compared to EOS dcSSc patients (Suppl. Fig. 1). No differences were observed in EGFL7 levels in the supernatants of cultured HC- and dcSSc-FBs (Suppl. Fig. 2).

## Discussion

In this study we show for the first time that EGFL7, a molecule so far considered to be endothelial-specific, is expressed in normal FBs and involved in the regulation of collagen expression and angiogenesis. Thus, during physiological conditions, EGFL7 may play a role in the homeostasis of ECM and the vasculature. We show that EGFL7 and its regulatory miR-126 are abnormally expressed in SSc, a disease in which alterations of ECM production and angiogenesis are strongly linked. In particular, EGFL7 expression is increased in EOS dcSSc skin and FBs when compared with HC. By contrast, EGFL7 levels were downregulated in the LSS dcSSc skin, despite being abundant in patient sera. These differences were reflected in the levels of the negative regulator of EGFL7 miR-126. In EOS SSc skin, increased EGFL7 expression may represent a homeostatic mechanism evolving to counteract the SSc-associated vasculopathy, whereas decreased expression in LSS dcSSc may suggest that after an early activation during the onset of the disease, progressive down-regulation may contribute to more severe vasculopathy such as that observed in LSS dcSSc (Fig. 1). Consistent with this notion, the defects in angiogenesis associated with both EOS and LSS dcSSc-FBs in our experimental system were reversed by treatment with exogenous rhEGFL7 (Fig. 7). Moreover, we found that higher levels of EGFL7 RNA were significantly associated with a lower MRSS in EOS dcSSc, supporting a reverse pathogenic correlation between EGFL7 and SSc (Suppl. Fig. 3). In addition, we show that EGFL7 promotes migration and invasion of SSc-FBs but not proliferation. On the other hand, while SSc-FBs, when co-cultured with HUVECs strongly impair the angiogenesis in an orgabnotypic co-culture system, this impairment is restored by exogenous EGFL7 stimulation. Taken together, these findings suggest that EGFL7 and its specific microRNA miR-126 may be integral parts of SSc pathogenesis.

It is widely accepted that FBs are involved both in physiological and pathological angiogenesis through two main mechanisms: the production of angiogenic factors, and matrix remodeling^5,18^. Recently, it has been shown that expression of members of the EGF-like family, such as EGFL6 and EGFL7, in bone cells can stimulate angiogenesis during bone remodeling^13,14^. We may therefore hypothesize that, mirroring what happens during bone remodeling, dermal FBs, may play a role in modulating the angiogenic process occurring in normal skin and during SSc via EGFL7 secretion. Regarding the differences observed in EOS and LSS dcSSc, several other studies have reported initial up-regulation of pro-angiogenic factors during early stages of SSc followed by a down-regulation in the later stages of the disease. Such reported factors are the intercellular adhesion molecule-1 expressed in peripheral blood mononuclear cells (PBMC) and skin FBs, junctional adhesion molecules expressed in dermal ECs and FBs, stromal derived factor (SDF)-1 in dermal ECs, endothelin-1 in monocytes and skin FBs, E-selectin, P-selectin and monocyte chemoattractant molecule-1/CC chemokine ligand 2 in the skin^31–34^. Therefore, EGFL7 may share the same fate as many other angiogenic factors during the course of SSc. As far as ECs are concerned, it has been shown that during reparative angiogenesis activated endothelium strongly up-regulates EGFL7, again supporting a pro-angiogenic role for EGFL7. A previous published study reported a decrease in EGFL7 expression levels in ECs both in early and late SSc consistent with the observed vasculopathy^35^. However in our study, we show clearly that the EGFL7 is still expressed in ECs from patients with EOS SSc, although its expression is not homogeneously distributed and cells can be detected that express lower levels or are even negative for EGFL7 expression (Fig. 1). This apparent discordance may be due to the new classification criteria of SSc^29^ applied in our study, which allowed us to consider in this study several patients who previously did not fulfill the 1980 classification criteria for SSc^36^.

During both physiological and pathological conditions FBs become activated and secrete different angiogenic molecules^18^. Since TGF-β, a key driver of SSc pathogenesis^2^, modulates VEGF expression thus modulating angiogenesis^37^, we further investigated its potential role in modulating EGFL7 expression. EGFL7 expression was strongly induced in culture in HC-FBs after TGF-β stimulation. In contrast, EOS SSc-FBs constitutively expressed higher EGFL7 RNA levels and any further stimulation by TGF-β did not change the expression levels. We may hypothesize that during the course of SSc, a pathological environment enriched in TGF-β may contribute to increased EGFL7 expression, thus influencing vascular remodeling, while at later stages other factors may contribute to a reduction of EGFL7 expression levels which in combination with the pro-fibrotic environment induced by TGF-β and induction of anti-angiogenic growth factors such as PEDF^31^ results in the severe vasculopathy observed at later stages of SSc.

The ECM is a key regulator of angiogenesis. It has been shown previously that ECM rigidity may promote sprouting and vascularisation of hypoxic tissues^5,15,16^. In our study treatment with rhEGFL7 decreased COL1A1 expression in SSc-FBs while EGFL7 silencing in SSc-FBs upregulated COL1A1 expression. Accordingly, we correlated the EGFL7 mRNA levels with the MRSS and we observed that patients with high EGFL7 levels exhibit a low MRSS, suggesting a possible role of this molecule in fibrosis. On the contrary, no effect was observed on the COL1A1 expression with treatment of HC-FBs suggesting that regulation of collagen expression by EGFL7 may be more specific to SSc. Studies have shown that EGFL7 transgenic mice exhibit a loose and non-resilient skin through inhibition of crosslinking of tropoelastin molecules into mature elastin polymers^11^, suggesting that EGFL7 may control ECM rigidity via different mechanisms in physiological conditions. FBs may be involved in vascular remodeling, under physiological and pathological conditions, by modifying the rigidity of ECM. EGFL7 may modulate angiogenesis via different mechanisms: on one hand, EGFL7 may promote cell adhesion, albeit weaker compared to cell adhesion to fibronectin and collagens, thus favoring EC motility^7^; on the other hand, transiently decreasing the collagen formation as shown in this study, thus modulating ECM rigidity. After EGFL7 silencing in SSc-FBs treated with TGF-β, we observed an increase in COL1A1 production compared to untreated SSc-FBs, suggesting that the modulation of COL1A1 expression in SSc is TGF-β dependent.

Furthermore, in this study we show that EGFL7 promotes fibroblast migration and invasion suggesting that EGFL7 may be involved in the recruitment of fibroblasts to the site of new vessels formation, further contributing to pro-angiogenic environment by secreting the angiogenic EGFL7 and by modulating ECM rigidity. Our results are in line with the available literature regarding the critical role of EGFL7 in regulating cell migration and invasion of FBs, ECs and various cancer cells, thus influencing processes such as wound healing, vascular remodeling and metastasis^7,38,39^. In early SSc we propose that EGFL7 plays a pro-angiogenic role suppressing vasculopathy which then increases at later stages when EGFL7 is absent for example in LSS dcSSc. Secreted EGFL7 in the sera of dcSSc patients was increased when compared to HC, while this increase was more prominent in the sera LSS dcSSc patients. This most probably reflects the fact that in EOS patients EGFL7 enters the blood circulation and is remotely distributed in various tissues, where may exert its role early on angionenesis^11^. In LSS dcSSc patients where blood vessels are largely absent, EGFL7 is not retained in the tissue, and thus it may remain at elevated levels in the sera. Surprisingly, despite detecting expression of EGFL7 by FBs in culture, EGFL7 was not detected in the supernatants of cultured FBs, suggesting that either fibroblasts may contribute minimally to EGFL7 secreted in the sera, and therefore only play a role in EGFL7 production locally, or secretion may be higher *in vivo*.

It has been shown recently that the EGFL7 gene contains the sequence for miR-126 which is predicted to negatively control EGFL7 expression. Mature miR-126 binds to a complementary sequence within EGFL7 mRNA and preventing translation thus, may act as a negative feedback mechanism^28^. Our results show that in EOS SSc-FBs the up-regulation of EGFL7 was associated with a down regulation of miR-126 expression. In contrast, in LSS SSc-FBs, which displayed normal levels of EGFL7 expression there were higher levels of miR-126 expression.

## Conclusions

We show that EGFL7, so far considered an endothelial-specific molecule, is abnormally expressed and regulated in normal and SSc FBs, and may influence the angiogenic process by modulating the expression of COL1A1 thus modifying the ECM. Moreover, miR-126, the negative regulator of EGFL7 gene is also abnormally and coordinately expressed in SSc. EGFL7 can exert pro-angiogenic effects both in early disease and at later stages when vasculopathy is more prominent. Taken together, these results suggest that a therapeutic strategy aimed to increase EGFL7 levels may present a valid approach to block the progressive vascular desertification observed in SSc, a condition for which specific pro-angiogenic therapies are still lacking.

## Methods

### Patients and controls

Full-thickness biopsy samples, 2 × 0.5 cm, isolated from excisional biopsy were obtained from clinically involved skin of one-third of the distal forearm of patients affected from the diffuse SSc according to LeRoy^40^. All patients fulfilled the 2013 classification criteria for SSc^29^. Skin with a Rodnan modified skin thickness score^41^ of ≥1 was considered to be clinically involved.

To be sure that 50% out of our patients were in a very early phase of SSc, considering that the terms “early”, at present, is referred to an undifferentiated connective tissue disease at higher risk to develop scleroderma, as suggested by the pivotal study of Koening *et al*.^42^ more than a time frame from the beginning of the disease, we further divided our patients in 2 subsets: patients fulfilling the classification criteria in less than one year from Raynaud’s Phenomenon onset (Early Onset Subset –EOS, n = 10), and all the others (Long Standing Subset –LSS, n = 10). Skin samples from the same region of 10 age- and sex-matched HC that underwent to a surgical treatment for trauma were used as control. The skin samples were processed for IHC and FB cell isolation and culture. All SSc patients underwent a 20-day washout from any immunosuppressive treatment and one month from intravenous prostanoids, before performing skin biopsy. During this period, only proton-pump inhibitors and clebopride were allowed. Patients who could not undergo therapeutic washout, due to severe organ complications, were not enrolled in the study. Biopsies were taken after all participants provided written informed consent in accordance with the declaration of Helsinki, and the study was approved by our local ethics committee (ASL Avezzano-Sulmona-L’Aquila, No. 015408/17). Demographic and clinical characteristics of the patients are shown in Table 1.

**Table 1:** Clinical features of 20 SSc patients.

SEX/AGE	Disease duration (years from RP) at skin biopsy	MRSS at skin biopsy	Autoantibodies	ILD	SRC/PAH	RP/Digital ulcers
F/43	<1	8	ANA/Scl-70	Yes	No/Yes	Yes/Yes
F/39	<1	9	ANA/Scl-70	No	No/No	Yes/No
F/31	<1	9	ANA/Scl-70	No	No/No	Yes/No
F/32	<1	8	ANA/Scl-70	No	No/No	Yes/Yes
F/45	<1	11	ANA/Scl-70	No	No/No	Yes/No
F/38	<1	9	ANA/Scl-70	Yes	No/Yes	Yes/No
F/44	<1	10	ANA/Scl-70	No	No/No	Yes/Yes
F/37	<1	11	ANA/Scl-70	No	No/No	Yes/No
F/49	<1	9	ANA/Scl-70	No	No/No	Yes/No
F/31	<1	9	ANA/Scl-70	No	No/No	Yes/Yes
F/56	4	21	ANA/Scl-70	Yes	No/No	Yes/No
F/51	6	16	ANA/Scl-70	No	No/No	Yes/Yes
F/47	6	10	ANA/Scl-70	No	No/No	Yes/No
F/54	7	14	ANA/Scl-70	No	No/No	Yes/No
F/54	5	21	ANA/Scl-70	No	No/Yes	Yes/No
F/61	5	19	ANA/Scl-70	No	No/No	Yes/No
F/64	4	17	ANA/Scl-70	No	No/No	Yes/No
F/67	5	19	ANA/Scl-70	No	No/No	Yes/No
F/49	5	23	ANA/Scl-70	Yes	No/No	Yes/No
F/59	7	26	ANA/Scl-70	No	No/No	Yes/No

RP: Raynaud’s phenomenon, MRSS: modified Rodnan skin score, ANA: antinuclear antibodies, Scl-70: anti topoisomerasi antibodies, ILD: interstizial lung disease, SRC: scleroderma renal crisis, PAH: Pulmonary Arterial Hypertension.

### Double Immunohistochemistry

Skin specimens were fixed in 10% buffered formalin, dehydrated in graded alcohol series, and embedded in paraffin. IHC analysis of human skin biopsy was performed on 3μm paraffin using a rabbit polyclonal EGFL7 antibody (Bioss), mouse monoclonal anti-CD34 antibody (Dako) and mouse monoclonal anti-aSMA (abcam). After antigen retrieval, the immunoreaction was detected using a goat anti-mouse-HRP and goat anti-rabbit-ALP (Menarini Diagnostics) followed by 3,3-diaminobenzidine tetrahydrochloride (DAB, Vector Laboratories) for CD34 and a-SMA visualization and Fast Red staining which produces a bright fuchsin-red fluorescence precipitate, for EGFL7 visualization (Menarini Diagnostics).

### Cell isolation and culture treatments

The skin specimen was placed into a 50-ml tube containing 10 ml of collagenase (Sigma) at 37 °C for 2 hrs. After digestion, the samples were cultured in Dulbecco modified Eagle’s medium (DMEM) (Sigma) supplemented with 10% fetal bovine serum (FBS; Standard South America origin), 100 units/ml penicillin, and 100 ng/ml streptomycin (Sigma) at 37 °C in a humidified atmosphere of 5%CO2. Third-passage FBs were analyzed for the surface expression of S100A4 antigen by flow cytometry (FACScan, Becton Dickinson) to assess their purity (S100A4+ different concentrations of rhEGFL7 (1–cells >99%).

We first examined the effect of rhTGF-β on EGFL7 expression and the effect of rhEGFL7 on COL1A1 expression on HC- and SSc-FBs, respectively. Cells were grown until 80–90% at confluence in a 6-well culture plate in DMEM with 10%FBS and subsequently starved in DMEM 1%FBS for 24 hrs and then stimulated with rhTGFβ (10 ng/ml) for 48hrs or different concentrations of rhEGFL7 (1–100 ng/ml; rhEGFL7, Abnova) for 24 or 48 hrs, respectively. The maximum concentration of EGFL7 used (100 ng/ml) was that previously reported to stimulate ICAM-1 expression and neutrophil adhesion to coronary artery endothelial cells^9^.

### qRT-PCR

Total RNA was isolated from both HC- and SSc-FBs treated and untreated, using Trizol reagent (Sigma). After determining the final concentration and quality of the obtained RNA using a NanoDrop spectrophotometer, 1ug of total RNA from each sample, was retrotrascribed in first-strand cDNA using the SuperScript III One Step RT PCR system (Invitrogen). For quantitative TaqMan real time evaluation of EGFL7 and Ki67, specific primers and probes were used, respectively (Hs00211952_m1; Hs01032443_m1; TaqMan Gene Expression assays, Applied Biosystems) while the 18 s gene was used as an internal control (Hs-03003631_g1, TaqMan Gene Expression assays, Applied Biosystems). For qRT-PCR evaluation of COL1A1, SYBR green kits were used and the primers were as follow: COL1A1 5′-CCTCCAGGGCTCCAACGAG-3′ (forward) and 5′-TCAATCACTGTCTTGCCCCA-3′ (reverse). Results were analyzed after 40 cycles of amplification using the ABI7500 Fast Real Time-PCR System and relative quantification was measured using the Comparative CT (Threshold cycle) Method.

### Western blot

For protein extraction from cells, confluent monolayers of HC- and SSc-FBs treated and untreated were washed with PBS and then scraped in ice-cold RIPA lysis buffer (Sigma). Total extracts were assayed for protein concentration using the bicinchoninic acid assay (ThermoScientific). Electrophoresis was performed using 35 ug for each sample through a 10%polyacrylamide gel and then transferred to PVDF membranes. After an overnight block with 5% non-fat dry milk, membranes were incubated with a goat polyclonal EGFL7 antibody (AF3638, R&D Systems) in TBS–0.1% Tween20 overnight at 4 °C. After incubation with horse peroxidase-conjugated anti-goat IgG (Thermofisher Scientific), immune complexes were detected with the enhanced chemiluminescence detection system (Amersham Biosciences). All the signals were quantified by normalizing to the β-actin signal. Furthermore, for the evaluation of the secreted COL1A1, equal volumes (30 µl) of cell culture supernatants (from equal amount of FBs) were also collected for use in WB using antibody to COL1A1 (Rockland Immunochemicals)^43,44^.

### Immunofluorescence (IF)

For IF on cultured isolated HC- and SSc-FBs cells were grown on 8-well culture slides (BD) maintained in DMEM medium (Sigma) supplemented with 10%FBS (Sigma) and 100 units/ml penicillin, and 100 ng/ml streptomycin (Sigma) at 37 °C in a humidified atmosphere of 5%CO_2_. FBs were fixed in 4% buffered paraformaldehyde for 20 min and permeabilized with 0.1% Triton X-100 in PBS. Non specific antibody binding was blocked with 10% casein in PBS for 20 min. All cells were incubated for 1 hr with a rabbit polyclonal anti-EGFL7 (Bioss) and mouse monoclonal α-SMA antibody (abcam). The immunoreaction was revealed using the appropriate biotinylated secondary antibodies and the signal was amplified with a streptavidin AlexaFluor 488 coniugate (Invitrogen). Cell nuclei were visualized using DAPI. Fluorescence was analyzed using an Olympus BX53 fluorescence microscope (Fig. 2C).

### Migration and invasion assay

To assess the possible effects of EGFL7 on cell migration and invasion, Transwell insert chambers were used. Briefly, for the migration assay, SSc- and HC-FBs were serum-starved overnight in DMEM prior to initiation of the experiment. The lower chamber were filled with with 1 ml of 1%DMEM containing EGFL7 or vehicle control. Cells were resuspended in 200 ml of 1%DMEM and added to the upper chamber. Cells were then incubated at 37 °C for 24 hrs to allow cell migration through the membrane. Media were removed from the upper chamber by wiping with a cotton swab and the cells that were migrate to the lower surface of the filter were fixed in 70% ethanol for 30 min and stained with 0.2% crystal violet for 10 min. Cell migration were measured by counting 5 randomly selected fields per filter under a light microscope.

For the invasion assay, SSc- and HC-FBs (3 × 10^5^ cells) were seeded into upper chambers pre-coated with Matrigel in medium containing 1%FBS and the subsequent steps were the same as those of the aforementioned migration assay up until the cells were stained with crystal violet. The number of cells invading the Matrigel were measured in 5 randomly selected fields using an inverted microscope.

### qRT-PCR analysis of miR-126

Total RNA, enriched of miRNAs was isolated from cultured HC- and SSc-FBs by using Trizol reagent (Sigma), according to the manufacturer’s instruction (Qiagen Inc). Single-stranded cDNA was synthesized from total RNA using specific miRNA primers for miR-126 (TaqMan MicroRNA Assay, Applied Biosystems) and the TaqMan MicroRNA Reverse Transcription Kit (Applied Biosystems). Specific single TaqMan miRNA assays (Ambion/Applied Biosystems) was used to measure the expression levels of miR-126 in a model 7500 real-time PCR system analyzer (Applied Biosystems). Expression of the U6B small nuclear RNA (RNU6B) served as endogenous control.

### RNA interference

For silencing of EGFL7 expression, SSc-FBs were transfected with Silencer Select EGFL7-siRNA (Life Technologies) or with Silencer Select Negative Control non-targeting siRNA (NT) (Life Technologies) using Lipofectamine™ 3000 (Life Technologies). Transfection was performed according to the manufacturer’s instructions. Briefly, SSc-FBs were plated at 1 × 10^4^ cells/cm^2^, 24 hrs prior to transfection. Cultures were incubated for 24 hrs with 25 pmol of siRNA in 2 mL of OptiMem. After incubation, plates were washed and cells were allowed to recover in normal growth conditions (10%DMEM) for 24 hrs post-transfection or in growth conditions (1% FBS) supplemented with TGF-β 10 ng/ml for 48 hrs post-transfection.

### Tube formation assay

We performed an organotypic co-culture assay using FBs isolated from skin of EOS- and LSS-SSc, and HUVECs to investigate the effects of SSc-FBs on tubule formation *in vitro*, as previsouly described^29^. Briefly, HC- or SSc-FBs were seeded at 2 × 10^4^ cells/well in a 24-well plate and grown until confluence for 7 days in complete DMEM medium. Subsequently, HUVECs were seeded on top of the FBs monolayer at 8.5 × 10^3^ cells/well in a 1:1 mixture of Human Large Vessel Endothelial Medium (TCS Cellworks) and complete DMEM medium (Sigma) supplemented with 10%FCS (Sigma) and rhEGFL7 100 ng/ml or VEGF 25 ng/ml, were added to the medium. Co-cultures were fixed for immunohistochemistry 5 days after HUVECs seeding. Fixation was carried out using ice-cold 70% ethanol for 30 min. ECs were successively labeled with mouse monoclonal anti-CD31 antibody^45^.

### ELISA

Secreted EGFL7 was detected by ELISA in HC and patients sera and supernatants from cultured FBs as described in supplementary material and methods.

### Statistical analysis

GraphPad Prism 5.0 software was used for statistical analyses. Results are expressed as mean ± SEM. Student’s t test was used as appropriate for analyses. Pearson correlation was used as appropriate for the correlation between EGFL7 levels and MRSS. *P* values less than 0.05 were considered significant.

## Supplementary information

Supplementary information and data
