# Supplementary material for: Epidermal Growth Factor Like-domain 7 and miR-126 are abnormally expressed in diffuse Systemic Sclerosis fibroblasts
Source: Sci Rep. 2019 Mar 14;9:4589. doi: 10.1038/s41598-019-39485-8 (PMC6418261; doi:10.1038/s41598-019-39485-8)
Supplement: Supplementary file 1 — Supplementary information and data [file 41598_2019_39485_MOESM1_ESM.pdf]

Supplementary Information for: Epidermal Growth Factor Like-domain 7 and miR-126 are abnormally expressed in diffuse Systemic Sclerosis fibroblasts

Vasiliki Liakouli[*1], Paola Cipriani[1], Paola Di Benedetto[1], Noemi Panzera[1], Piero Ruscitti, Ilenia Pantano[1], Onorina Berardicurti[1], Francesco Carubbi[1], Filomena Esteves[2], Georgia Mavria[3], Francesco Del Galdo[4], Roberto Giacomelli[1]

Affiliation

[1*]Department of Biotechnological and Applied Clinical Science, Rheumatology Unit, School of Medicine, University of L'Aquila, Delta 6 Building, Via dell'Ospedale, 67100, L'Aquila, Italy

[2*]Leeds Institute of Cancer and Pathology, University of Leeds, Leeds, United Kingdom.

[3*]Signal Transduction and Tumor Microenvironment Group, Leeds Institute of Cancer and Pathology, University of Leeds, Leeds, United Kingdom

[4*] Division of Rheumatic and Musculoskeletal Diseases, Leeds Institute of Molecular Medicine, University of Leeds, Leeds, United Kingdom

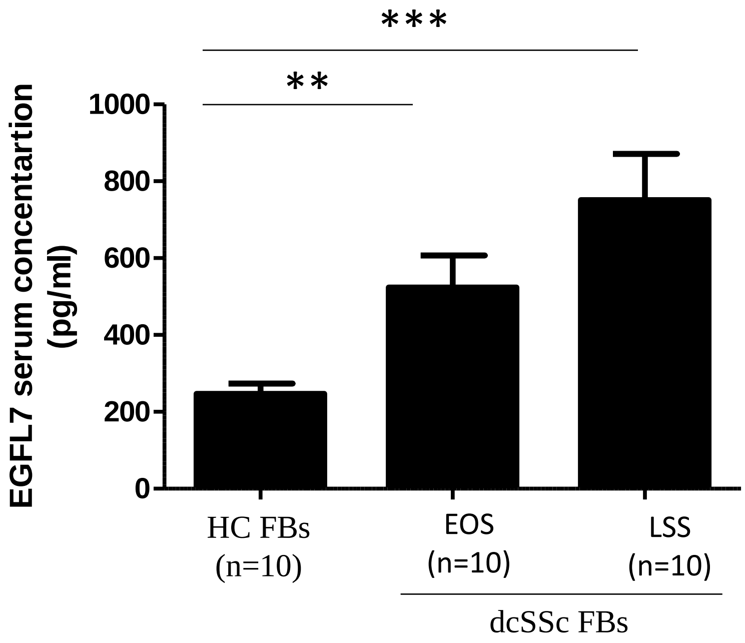

Supplementary Figure 1: EGFL7 serum levels are increased in the EOS and LSS dcSSc patients.

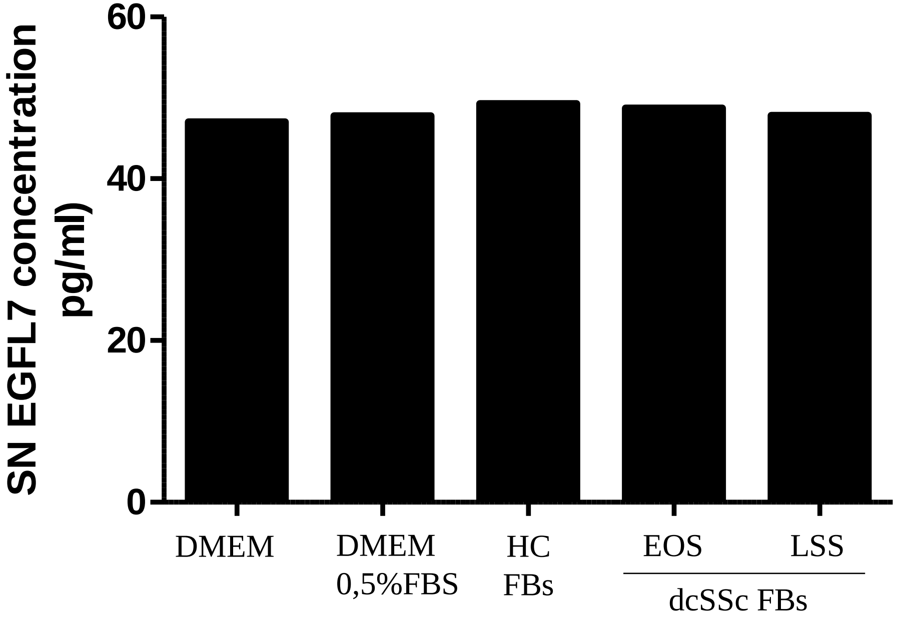

Supplementary Figure 2: No differences were observed in the secreted EGFL7 in the supernatant of cultured HC- and dcSSc-FBs

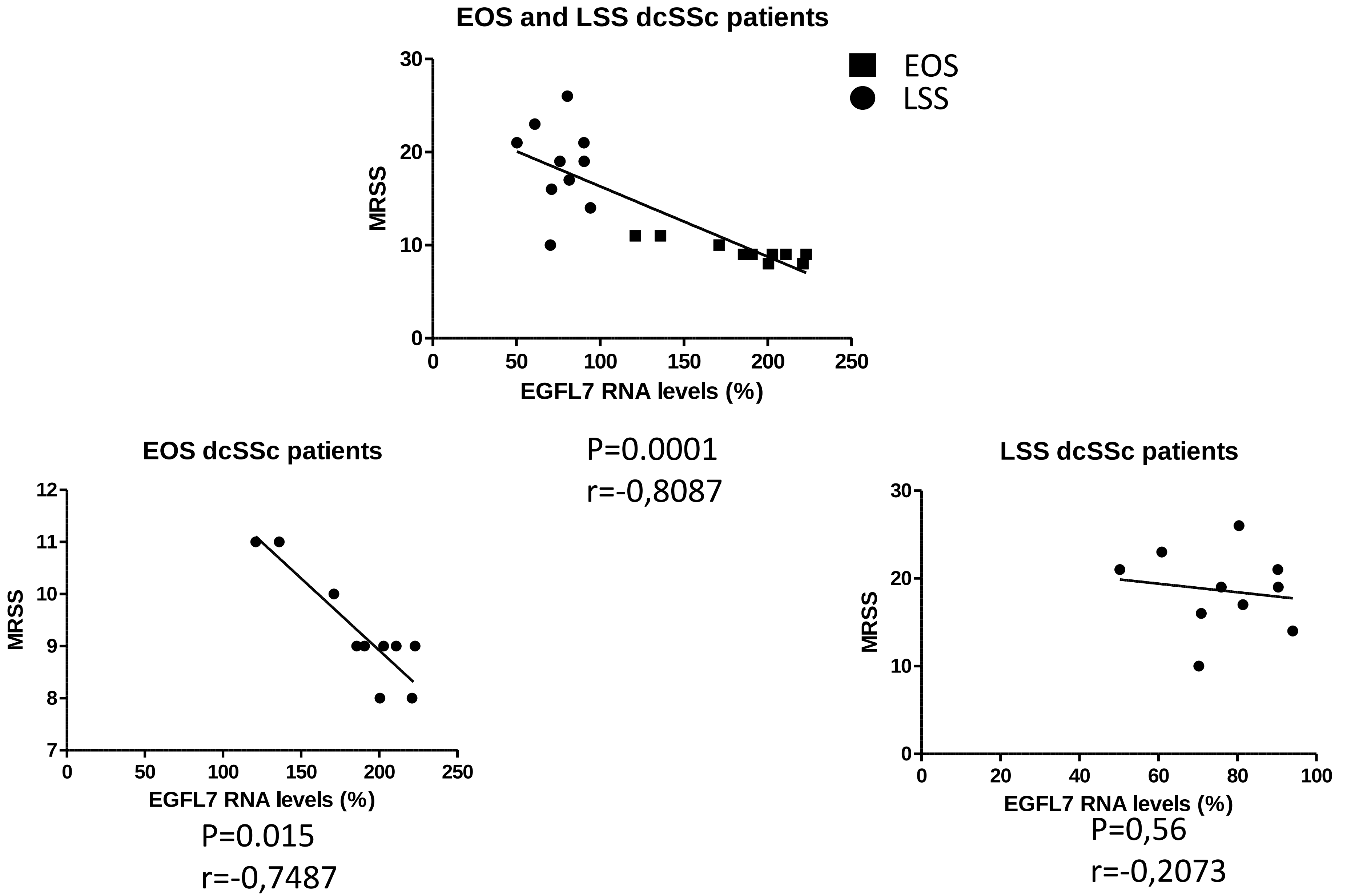

Supplementary Figure 3: Negative correlation between EGFL7 RNA levels and the MRSS of EOS and LSS dcSSc patients.

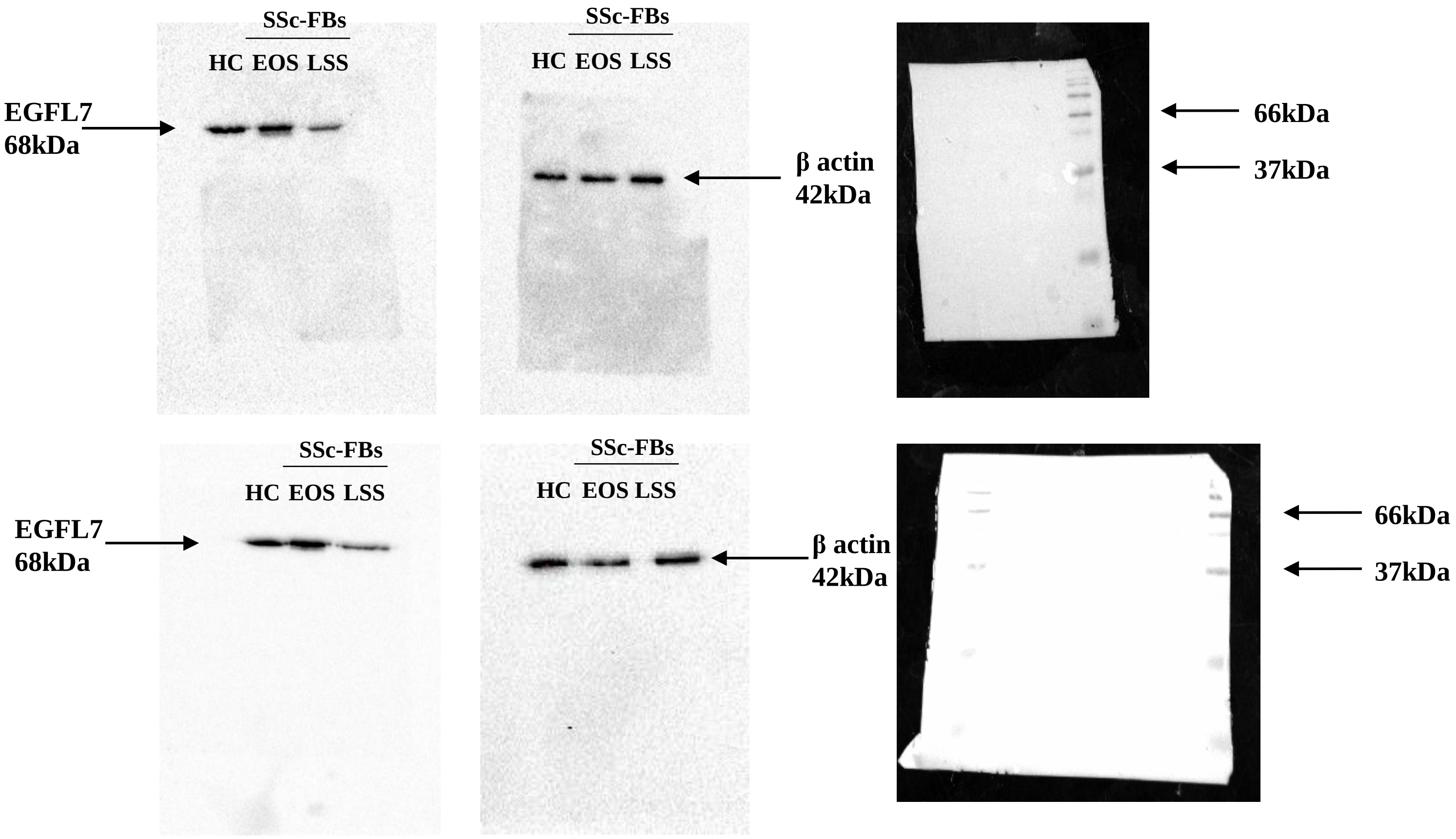

Supplementary Figure 4: Original blot for Figure 2B

SUPPLEMENTARY MATERIAL AND METHODS

Enzime-linked aptamer sorbent assay (ELISA)

The amounts of EGFL7 released in the supernatant were determined by using specific Quantikine Human immunoassay ELISA kit (My Biosource), according to the manufacturer's protocol. For circulating levels of EGFL7, sera were collected from HC, EOS and LSS dcSSc patients and tested by the same ELISA assay used for FBs culture. For secreted EGFL7 levels on supernatant, cells were grown until 80-90% at confluence in a 6-well culture plate in DMEM with 10%FBS and subsequently starved in DMEM 1%FBS for 24hrs and then cultured for 48hrs. Supernatant was then collected, centrifuged and tested by the ELISA assay.

SUPPLEMENTARY FIGURE LEGEND

Suppl. Fig. 1: Serum EGFL7 is increased in EOS and LSS dcSSc patients. Secreted EGFL7 in the sera of EOS (n=10) and LSS (n=10) dcSSc patients is increased when compared to HC (n=10) (p=0.0076 and p=0.0006, respectively) and this increase is more prominent in the LSS-dcSSc patients, most probably reflecting the fact that in the EOS patients, EGFL7 enters the blood circulation and is remotely distributed in various tissues, where may exert its role early on angionenesis. However, no statistically differences were found between the EOS and LSS dcSSc group (p=0.18).

Suppl. Fig 2: SN EGFL7 in HC- and dcSSc-FBs. No differences were observed in the secreted EGFL7 in the supernatant of cultured HC- and dcSSc-FBs (p=0.10 and p=0.076, respectively).

Suppl. Fig. 3: Negative correlation between EGFL RNA levels and the modified Rodnan skin score (MRSS) of EOS and LSS dcSSc patients. Pearson correlation demonstrated that there is a negative correlation between EGFL7 RNA levels and the MRSS. In particular, patients with increased levels of EGFL7 RNA they have a lower MRSS while patients with low levels of EGFL RNA they have a higher MRSS (p=0.0001; r=-0,8087).
